# Phase-change perovskite metasurfaces for dynamic color tuning

**DOI:** 10.1515/nanoph-2022-0143

**Published:** 2022-06-06

**Authors:** Jingyi Tian, Daniele Cortecchia, Yutao Wang, Hailong Liu, Elena Feltri, Hong Liu, Giorgio Adamo, Cesare Soci

**Affiliations:** Centre for Disruptive Photonic Technologies, TPI, Nanyang Technological University, 21 Nanyang Link, Singapore 63737, Singapore; Division of Physics and Applied Physics, School of Physical and Mathematical Sciences, Nanyang Technological University, 21 Nanyang Link, Singapore 637371, Singapore; Centre for Nano Science and Technology (CNST@PoliMi), Istituto Italiano di Tecnologia, Milan 20133, Italy; Institute of Materials Research and Engineering Agency for Science, Technology and Research (A*STAR) 2 Fusionopolis Way, Singapore 138634, Singapore; Department of Physics, Politecnico di Milano, Piazza Leonardo da Vinci 32, Milano 20133, Italy

**Keywords:** dynamic color tuning, halide perovskite, metasurfaces, phase-change

## Abstract

Halide perovskite metasurfaces are attracting increasing interest for applications in light-emitting and display technologies. To access the wide range of colors required for these applications, the main mechanism exploited thus far has been chemical engineering of the perovskite compounds – this constitutes a significant limitation for the dynamic switching of optical response desirable in actual devices. Here we demonstrate polarization-dependent, dynamic control of structural color and emission wavelength in an all-dielectric phase-change halide perovskite nanograting metasurface, by temperature tuning. This is underpinned by the significant change in the perovskite optical constants which accompanies its phase-transition around room temperature. The functionalities demonstrated in our work bearing potential for applications in light-emitting devices, displays and spatial-light-modulators.

## 1 Introduction

Optical metasurfaces [1], two dimensional arrays of nanostructures with subwavelength thickness which strongly confine light at the nanoscale, allow arbitrary manipulation of amplitude [2], polarization [3, 4] and phase [5] of light, enabling a variety of optical functionalities on demand [1, 6, 7]. Halide perovskites have recently emerged as a solution-processable platform of choice for the realization of all-dielectric active metasurfaces that generate a wide range of colors [8–10], produce multifold enhancement of light emission [9, 11, 12] and lasing [13–16], thanks to their outstanding luminescence properties, high refractive index, ease of processing and low cost [17, 18]. These properties make halide perovskite metasurfaces excellent candidates for applications in light-emitting diodes [19, 20], color display [8–10] and microlasers [21]. In many of these applications, however, dynamic tunability of colors is a highly desirable requirement beside the access to a wide gamut. So far, the main mechanism used to vary the color response in halide perovskites is the alteration of their chemical composition [10], an approach that is viable only for static device applications. Dynamic ion exchange approaches have also been demonstrated, but with somewhat limited applicability [22].

As a matter of fact, halide perovskites sustain a wide range of crystallographic phases [18], determined by chemical composition [23], pressure [24] and temperature [25], which can result in significant variations in the optical constants and the emission spectra [26]. While demonstrations of phase-change tunable perovskite metasurface emitters [27] and microlasers [28] have recently appeared, they operated at cryogenic temperatures (tetragonal-orthorhombic structural phase transition around 130–160 K in MAPbI_3_), which poses serious operational limitations – fortunately halide perovskites that experience phase transition close to room temperature have lately been discovered [29].

Here we demonstrate an all-dielectric metasurface, based on a simple nanograting design, using a halide perovskite which undergoes a phase transition around room temperature. We use the significant change in the optical constants associated to the perovskite phase transition to engineer a polarization-dependent dynamic color tunability controlled by temperature. Given the large color gamut accessible by nanostructuring, the associated color change – well distinct from that of the unstructured perovskite films – and its tunability close to room temperature, this demonstration paves the way to the realization of dynamic light-emitting devices, displays and spatial-light-modulators that could potentially operate by thermoelectric cooling.

## 2 Results and discussion

**Figure 1:**
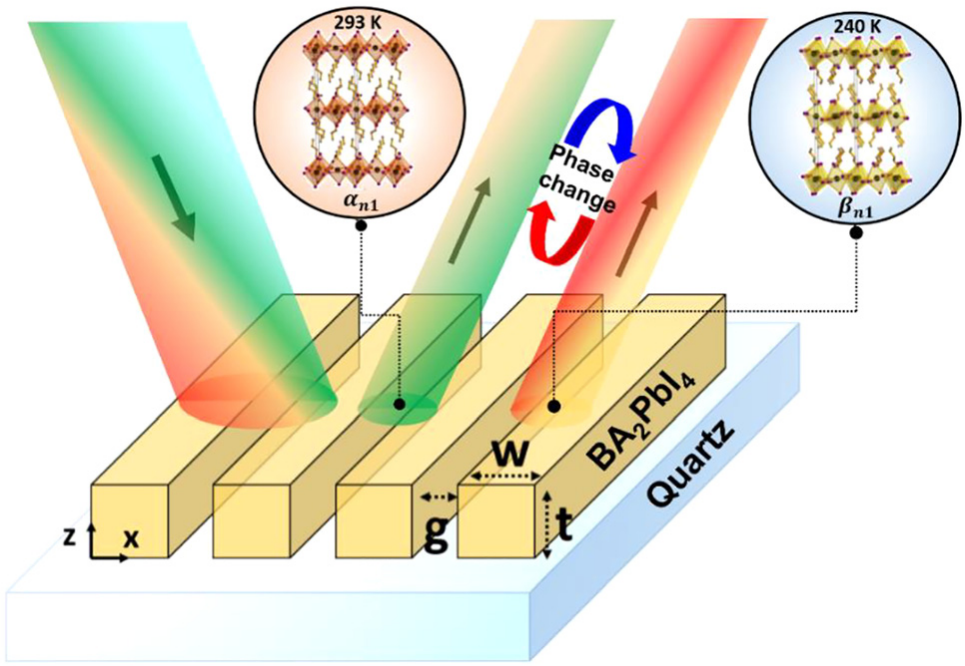
Phase-change perovskite metasurface with dynamic color tunability. Schematic of a tunable phase-change BA_2_PbI_4_ perovskite nanograting metasurface on quartz substrate. The reflected color is dynamically controlled through the switching of crystallographic phases of the perovskite by altering the ambient temperature.

**Figure 2:**
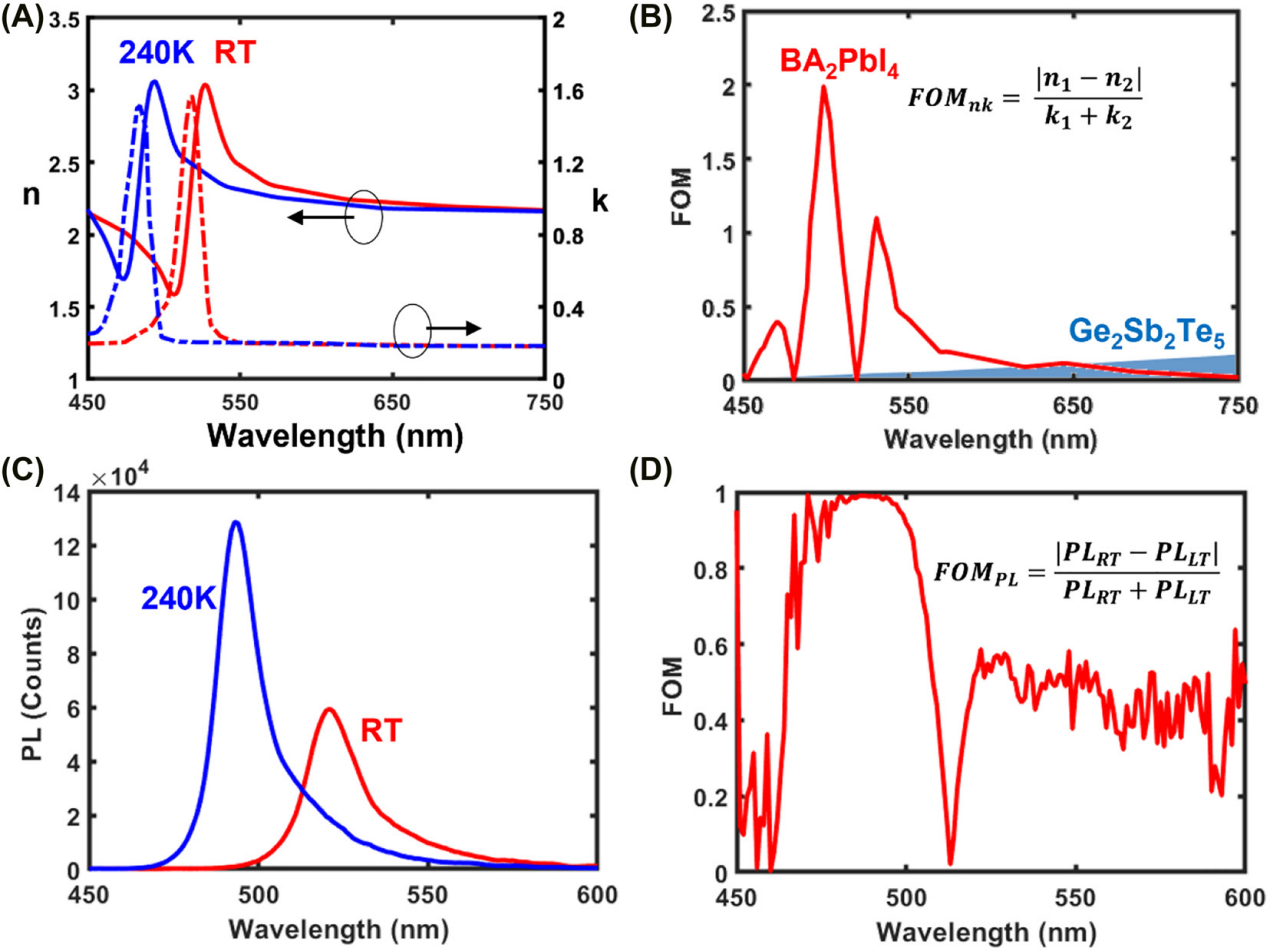
Optical properties and switching performances of BA_2_PbI_4_. (A) Real *n* (full lines) and imaginary *k* (dashed lines) parts of the refractive index of BA_2_PbI_4_ at 293 K (red curves) and 240 K (blue curves). (B) Comparison between the phase-change optical constants figure of merit (FOM_PC_) of BA_2_PbI_4_ and Ge_2_Sb_2_Te_5_ in the visible spectrum. (C) Photoluminescence spectra at 293 K (red curves) and 240 K (blue curves). (D) Phase-change photoluminescence figure of merit (FOM_PL_) of BA_2_PbI_4_.

To realize the phase-change nanograting metasurface, the butylammonium lead iodide perovskite of the Ruddlesden–Popper series, BA_2_PbI_4_, was selected due to its significant change in refractive index upon phase transition near room temperature between two orthorhombic phases *α*_*n*1_ and *β*_*n*1_, respectively, at 240.5 K (*α*_*n*1_ → *β*_*n*1_) upon cooling and 270.5 (*α*_*n*1_ → *β*_*n*1_) upon heating [29]. The change in refractive index is expected to induce a change in the spectra of light reflected and transmitted by nanograting metasurface carved on BA_2_PbI_4_ films span-cast on quartz substrates, as exemplified in the schematic of Figure 1. The experimental optical constants of the BA_2_PbI_4_ films, whose thickness is estimated by atomic force microscopy (Figure S1), are retrieved by measuring their reflection and transmission spectra at the two representative temperatures of 293 and 240 K and applying Kramers–Kronig relations (Figure 2A). The agreement between the calculated and the measured reflection spectra from a BA_2_PbI_4_ film at different temperatures is illustrated in Figure S2.

A phase-change figure of merit for BA_2_PbI_4_ can be defined as ${FOM}_{PC}=\left|\Delta n\right|/\left(k_{1}+k_{2}\right)$ , where Δ*n* denotes the change of refractive index and *k*_1_ + *k*_2_ is the sum of extinction coefficients of the two phases that account for optical losses [30]. The FOM_PC_ for BA_2_PbI_4_ is higher than the FOM for the canonical phase-change chalcogenide Ge_2_Sb_2_Te_5_, across a significant portion of the visible spectrum, peaking at *λ* = 500 nm, where it outperforms by almost two order of magnitude (Figure 2B) [31].

Like the refractive index, the photoluminescence (PL) spectra of BA_2_PbI_4_ change dramatically upon phase transition: the very clear green light emission (peaking at *λ* = 525 nm) observed at room temperature when the film is in the *α*_*n*1_ phase, undergoes a substantial blueshift to become a very pure cyan (peaking at *λ* = 490 nm) when the film is in the *β*_*n*1_ phase (Figure 2C). It is possible to define a photoluminescence change figure of merit of BA_2_PbI_4_ as ${FOM}_{PL}=\frac{\left|{PL}_{RT}-{PL}_{LT}\right|}{{PL}_{RT}+{PL}_{LT}}$ , which reaches 1 between 477–498 nm, corresponding to a distinct contrast between room-temperature PL and low-temperature PL.

**Figure 3:**
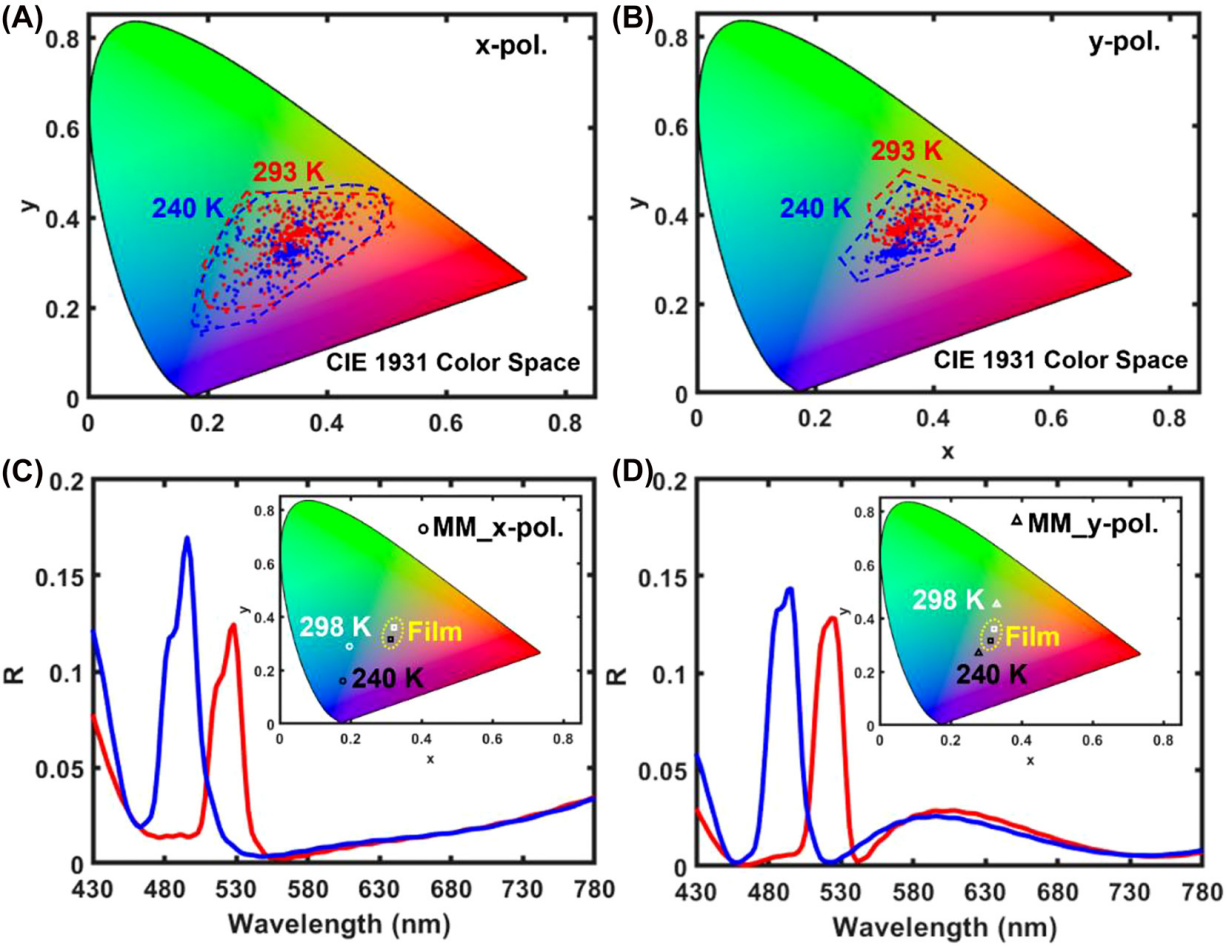
Large gamut and switching of colors with a BA_2_PbI_4_ nanograting metasurface. (A and B) Small changes in the geometrical parameters of the nanogratings, such as gap size (*g* = 50–200 nm), beam width (*w* = 50–350 nm) and milling depth (*t*_m_ = 0–240 nm) allow to access a large color gamut under, respectively, *x*-polarized and *y*-polarized incident light, thus significantly expanding on the color of the unpatterned BA_2_PbI_4_ perovskite film. (C and D) Numerically calculated reflection spectra of two BA_2_PbI_4_ perovskite metasurfaces under, respectively, *x*-polarized and *y*-polarized incident light, which show a large dynamically controllable color contrast variations of the temperature between 293 and 240 K.

The optical response of the BA_2_PbI_4_ metasurfaces can be tailored by adjusting the nanograting parameters indicated in Figure 1, namely gap size, *g*, beam width, *w*, and milling depth, *t*_m_. This large parameter space together with the additional degree of freedom given by the anisotropy of the design, allows access to a very large color gamut. Figure 3A and B exemplify the increase in color availability brought by the nanograting metasurfaces compared to the flat film. The simulated colors reflected by the metasurfaces are shown in the CIE 1931 maps [32] for nanograting geometrical parameters varying in the following ranges: *g* = 50–200 nm, *w* = 50–350 nm and *t*_m_ = 0–240 nm. A very rich reflection spectral response manifests itself in a very broad range of colors covering violet-blue to green and red for incident light with orthogonal polarizations at both room and low temperatures (the corresponding transmitted colors are shown in Figure S3).

Full wave electromagnetic FDTD simulations show that the optical response of the metasurfaces can be dynamically controlled with small variations in the temperature. In Figure 3C, an *x*-polarized plane wave, incident on a BA_2_PbI_4_ nanograting with *w* = 50 nm, *g* = 50 nm, *t*_m_ = 120 nm, carved on a 240 nm thin film, induces an optical mode confined between the perovskite ridges (Figure S4A), whose spectrum is significantly blue-shifted when the temperature of the perovskite film is reduced from 293 K (red curve) to 240 K (blue curve). The corresponding color reflected by the metasurface (circles in Figure 3C inset) switches from cyan to blue while it remains of a yellow hue for the un-patterned film (squares in Figure 3C inset). Another example is shown in Figure 3D for *y*-polarized light incident on a nanograting with *w* = 150 nm, *g* = 50 nm, *t*_m_ = 95 nm, where a similar blue-shift of the optical mode confined within the perovskite ridges (Figure S4B) takes place when the temperature is reduced from 293 to 240 K. The corresponding color reflected by the metasurface is switched from green to purple (triangles in Figure 3D inset).

**Figure 4:**
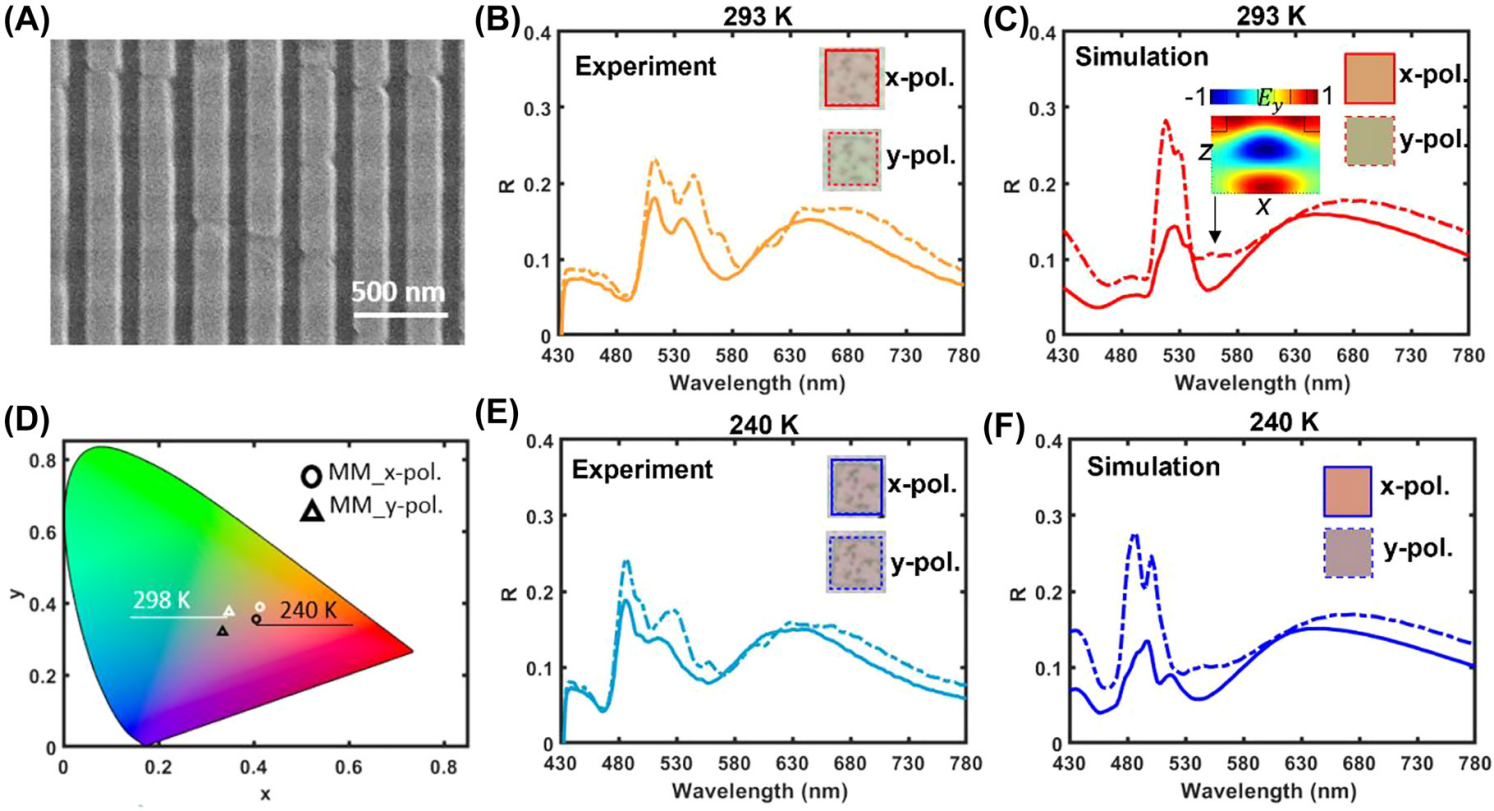
Experimental realization of a BA_2_PbI_4_ nanograting metasurface with dynamic color switching. (A) SEM image of a BA_2_PbI_4_ nanograting metasurface patterned by nanoimprint lithography. (B and E) Experimental *x*-polarized and *y*-polarized reflection spectra at, respectively, 293 and 240 K. The insets show the reflected color recorded by a CCD. (D) Reconstructed reflected colors of the metasurface in the color space based on the simulated reflection spectra for both polarizations at (C) 293 K and (F) 240 K.

The experimental realization of a BA_2_PbI_4_ perovskite metasurface with dynamic color tunability was done by milling nanograting metasurfaces (*w* ∼ 220 nm, *p* ∼ 320 nm and *t*_m_ ∼ 50 nm) in arrays of 50 × 50 μm area, through nanoimprint lithography on BA_2_PbI_4_ perovskite thin films of ∼240 nm thickness span-cast on quartz substrates (SEM image of the fabricated nanograting is shown in Figure 4A). The reflection spectra of the metasurfaces were measured under normal incidence across the entire visible region, for light polarized both parallel (*y*-polarized) and orthogonal (*x*-polarized) to the grating, at both 293 and 240 K. The subwavelength structuring of the films induced in the spectra clearly observable resonances, created by the interplay between the thin film interference and the grating modes at both room (Figure 4B) and low temperature (Figure 4E). In experiments, the color reflected by the metasurface under x-polarized light illumination barely changes through the phase transition (CCD-recorded colors in Figure 4B and E insets). However, the color reflected by the metasurface under *y*-polarized illumination changes from green at room temperature to cameo brown at low temperature (CCD-recorded colors in Figure 4B and E insets). Both the spectral and color response obtained via full wave electromagnetic FDTD simulations confirm the experimental observation at both room (Figure 4C) and low temperature (Figure 4F). The colors retrieved by the simulated spectra (insets of Figure 4C and F) are in good accordance with the experimental results at normal incidence. The diffraction pattern of the nanograting is expected to follow the angular dispersion of the resonances in the two polarizations, as shown by the simulated angle-resolved reflection maps in Figure S5. The more marked color change observed under y-polarized with respect to *x*-polarized illumination, for the fabricated metasurfaces, is confirmed by their *x*–*y* coordinate in the CIE 1931 color space (Figure 4D). The resonance excited around 540 nm under *y*-polarized illumination at room temperature is shown in the inset of Figure 4C; the resonance undergoes a blue shift around 530 nm when the temperature is lowered to 240 K.

**Figure 5:**
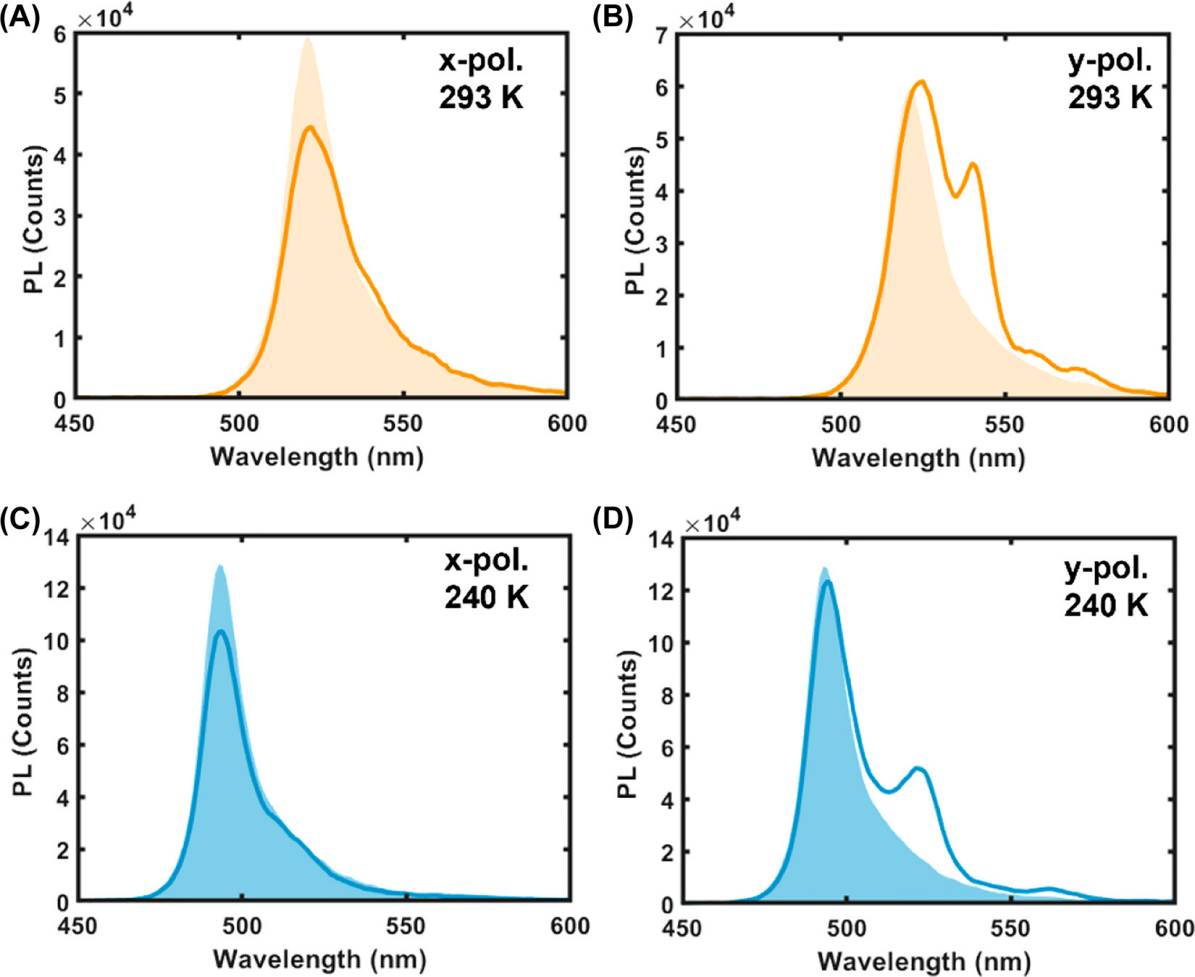
Polarization-dependent photoluminescence switching in BA_2_PbI_4_ nanograting metasurface upon phase transition. (A) *x*-polarized and (B) *y*-polarized PL from the BA_2_PbI_4_ nanograting metasurface at 293 K (orange curves): the orange-shaded areas are the PL spectra from the unstructured film at the same temperature. (C) *x*-polarized and (D) *y*-polarized PL from the BA_2_PbI_4_ nanograting metasurface at 240 K (light blue curves): the blue-shaded areas are the PL spectra from the unstructured film at the same temperature.

Beside the dynamic structural color tuning, the metasurface design can also be used to tune the PL emission of the perovskites. Whereas the *x*-polarized PL of the BA_2_PbI_4_ film (orange/blue shaded spectra in Figure 5A and C) is barely affected in intensity by structuring with a nanograting metasurface (orange and blue curves in Figure 5A and C), a distinct side peak appears in the *y*-polarized PL of the metasurface (orange and blue curves in Figure 5B and D). The additional peak occurs at *λ* = 540 nm, inducing a 4.5-fold enhancement in the PL emission at the temperature of 293 K (Figure 5B), and blue-shifts to *λ* = 530, associated to a 5-fold PL enhancement at 240 K nm (Figure 5D), upon phase transition of the BA_2_PbI_4_ film. This is a clear manifestation of the Purcell effect induced by the resonant mode (inset of Figure 4E) confined within the dielectric beams of the nanograting metasurface, which act as nanocavity.

## 3 Conclusions

In summary, we show that nanostructuring of perovskite films with metasurface designs, in combination with the temperature-inducing intrinsic crystallographic phase transitions, provides a simple and promising mechanism to actively control the optical response of the metasurface. By identifying a phase-change perovskite (BA_2_PbI_4_) which undergoes phase-transition and significant refractive index change around room temperature, we have experimentally realized a reconfigurable dielectric perovskite metasurface whose optical response can be tailored across a broad color gamut and achieved dynamic control of structural color and emission wavelength. With this novel approach for active tuning of the optical response of halide perovskites, beyond chemical synthesis, we expand the relatively limited library of phase change materials for the realization of active metadevices in the visible part of the spectrum. The functionalities demonstrated in our work bear potential for applications in light-emitting devices, displays and spatial-light-modulators.

## 4 Experimental section/methods

***Perovskite film preparation*** *:* Quartz substrates are immersed in a mixture of 2 ml Hellmanex II (Hellma Analytics) and 200 ml of DI water at 353 K for 10 min, rinsed in DI water, dried under nitrogen flow and cleaned by oxygen plasma. For the synthesis of the BA_2_PbI_4_ perovskite, the following steps are followed: (i) BAI (Dyesol) and PbI_2_ (99.99%, TCI) powders with a molar ratio of 2:1 are added into a mixed solvent of anhydrous dimethylformamide (DMF, Sigma-Aldrich) and dimethyl sulfoxide (DMSO, Sigma-Aldrich) with a volume ratio of 3:1; (ii) the mixture is stirred for 1 h at room temperature in a N_2_ atmosphere to form a 0.5 M solution, followed by filtering with a poly(vinylidene fluoride) (PVDF) syringe filter (0.45 μm). The films are deposited by spin-coating the solution on the quartz substrates at 5600 rpm for 35 s while dripping toluene 5 s after starting the spinning and then annealed on a hot plate at 373 K for 15 min. The entire precursor preparation and spin coating process are processed in a N_2_-filled glovebox.

***Thermal nanoimprint lithography:*** A negative resist (Hydrogen silsesquioxane, XR-1541-006) with the thickness of 170 nm is spin-coated on a silicon substrate at a speed of 1500 rpm for 1 min. E-beam lithography (ELS-7000 (Elionix Inc.)) is utilized to fabricate the nanograting metasurface under an acceleration voltage of 100 kV and the dose of 7600 μC/cm^2^. Inductively coupled plasma etching with a recipe of HBr (50 sccm) and O_2_ (3 sccm) gases at 5 mTorr is then employed to etch the Si substrate. Master molds are obtained after removing the HSQ mask in a buffered hydrofluoric acid. A nanoimprinter (Obducat NIL-60-SS-UV-Nano-imprinter) is used to transfer the metasurface from master mold to BA_2_PbI_4_ film at 30 bar and 90 °C. The imprinting time is optimized to be 30 min. At last, the imprinted sample is cooled down to room temperature and demolded from the master mold.

***Numerical simulations*** *:* The reflection and transmission spectra of perovskite metasurfaces are calculated using Lumerical FDTD. Periodic boundary conditions are adopted in lateral (*x*, *y*) directions and perfectly matched layers (PML) are constructed along the incident direction (*z*). The reconstructed colors from the spectra are based on CIE 1931 Color Space.

## Data and materials availability

The authors declare that all data supporting the findings of this study are available within this article and its supplementary material and are openly available in the NTU research data repository DR-NTU (Data) at https://doi.org/10.21979/N9/HA5YVM. Additional data related to this paper may be requested from the authors.

## Supplementary Material

Supplementary Material Details
