# Supplementary material for: Phase-change perovskite metasurfaces for dynamic color tuning
Source: Nanophotonics. 2022 Jun 6;11(17):3961–8. doi: 10.1515/nanoph-2022-0143 (PMC11502028; doi:10.1515/nanoph-2022-0143)
Supplement: Supplementary file 1 — Supplementary Material Details [file j_nanoph-2022-0143_suppl.pdf]

Supplementary Material

Phase-change perovskite metasurfaces for dynamic color tuning

Jingyi Tian[1,2], Daniele Cortecchia[3], Yutao Wang[1,2], Hailong Liu[4], Elena Feltri[1,2,5], Hong Liu[4], Giorgio Adamo[1,2]*, Cesare Soci[1,2]*

[1] Centre for Disruptive Photonic Technologies, TPI, Nanyang Technological University, 21 Nanyang Link, Singapore 63737, Singapore

[2] Division of Physics and Applied Physics, School of Physical and Mathematical Sciences, Nanyang Technological University, 21 Nanyang Link, Singapore 637371, Singapore

[3] Centre for Nano Science and Technology (CNST@PoliMi), Istituto Italiano di Tecnologia, Milan 20133, Italy

*[4] Institute of Materials Research and Engineering Agency for Science, Technology and Research (A*STAR) 2 Fusionopolis Way, Singapore 138634, Singapore*

[5] Department of Physics, Politecnico di Milano, Piazza Leonardo da Vinci 32, 20133 Milano, Italy

*Correspondence to: csoci@ntu.edu.sg, g.adamo@ntu.edu.sg

I THICKNESS AND REFLECTION SPECTRA OFA BA_2PBI_4 FILM

The AFM image of a BA_2PbI_4 film with a thickness of 240 nm is shown in Fig. S1, where the roughness is estimated to be around 10 nm.

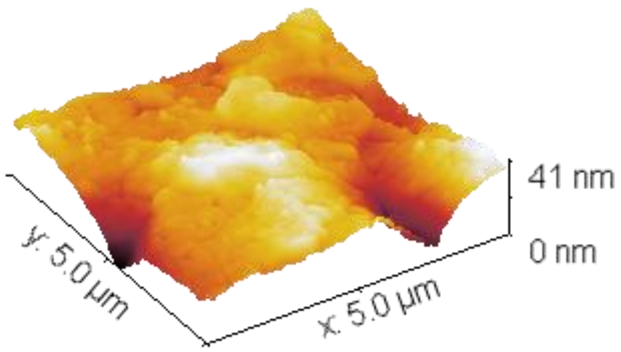

Fig. S1 AFM image of a BA_2PbI_4 film with thickness of 240 nm

The calculated and measured reflection spectra from a BA_2PbI_4 film at different temperatures are illustrated in Fig. S2, to prove the validity of the retrieved optical constants. Note that the optical constant of layered perovskites have strong anisotropy between the in-plane and out-of-plane directions, relative to the substrate. Since 2D perovskites obtained by long time spin-coating have randomly oriented domains within the plane of the substrate [Nature communications 9.1 (2018): 1-7.], we have adopted a simple isotropic model of the optical constants which accounts for the main features of the experiments. If necessary, the out-of-plane anisotropy can be compensated by variation in the thickness of the film and scaling of the nanograting structures.

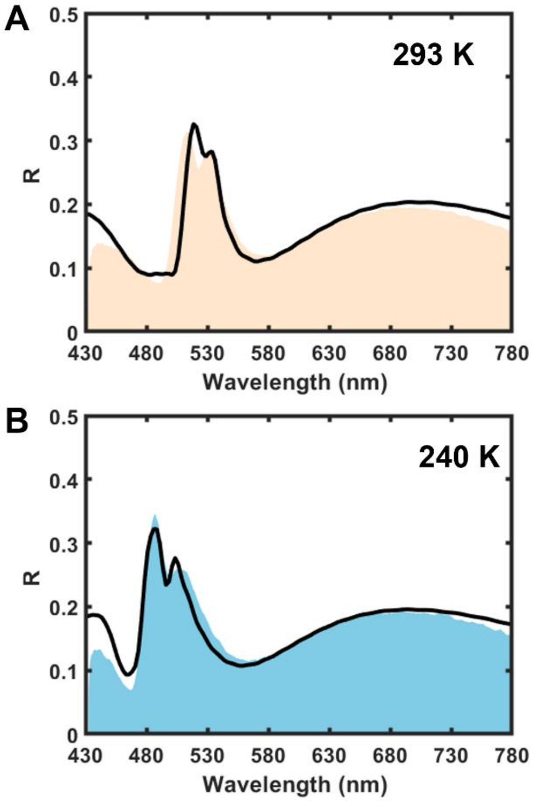

Fig. S2 Calculated (solid curves) and measured (shaded areas) reflection spectra from a 240nm thick BA_2PbI_4 film at different temperatures

II THE CALCULATED TRANSMITTED COLORS OF THE BA_2PBI_4 METASURFACE

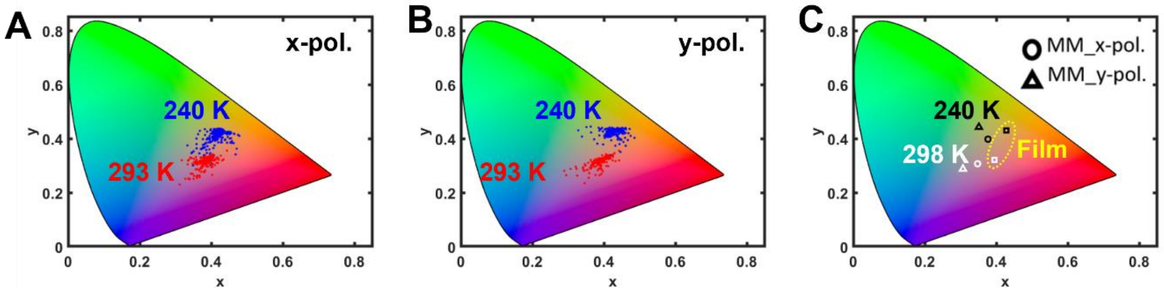

Fig. S3 (A-B) Accessible transmission color gamut under, respectively, *x*-polarized and *y*-polarized light incident on BA_2PbI_4 nanograting metasurfaces for variation of the grating geometrical parameters such as gap size (*g* =50-200 nm), beam width (*w* =50-350 nm) and milling depth (t_m =0-240 nm) (C) Numerically calculated transmission spectra of the same two BA_2PbI_4 perovskite metasurfaces of Fig 3C-3D under *x*-polarized and *y*-polarized incident light for variation of the temperature between 293 K and 240 K.

III OPTICAL RESONANCES OF THE BA_2PBI_4 METASURFACE

An x-polarized plane wave, incident on a BA_2PbI_4 nanograting carved on a 240 nm thin film with w = 50 nm, g = 50 nm, t_m = 120 nm, induces an optical mode confined between the perovskite ridges (Fig. S4A). Another example is illustrated in Fig. 3D for y-polarized incident light on the nanograting with w = 150 nm, g = 50 nm, t_m = 95 nm, where the optical mode confined within the perovskite ridges (Fig. S4B)

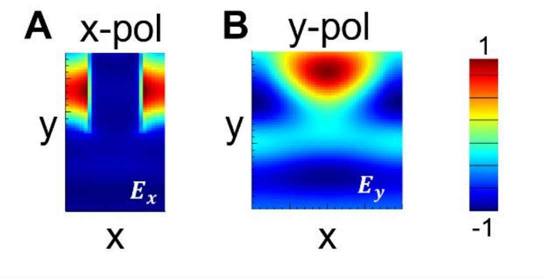

Fig. S4 Simulated electric field distributions of two illustrative metasurfaces with small variations of the temperature under (A) *x*-polarized and (B) *y*-polarized illumination.

IV ANGLE RESOLVED REFLECTION SPECTRA OF THE METASURFACE

The calculated angle-resolved reflection maps at room temperature and 240 K for both x- and y-polarized illuminations are shown in Fig. S5. The green arrows indicate the normal incidence resonance excited around 540 nm under y-polarized illumination at room temperature, which undergoes a blue shift to approximately 530 nm when the temperature is lowered to 240 K. As expected, the y-polarized resonance induced by the nanograting shows a strongly dispersive behaviour indicated by the dashed green line at angles away from the normal. This cannot be seen in the x-polarization maps where the nanograting does not produce resonances. Conversely, the dispersionless flat band near 520 nm (490 nm) at room temperature (240K) can be attributed to the intrinsic excitonic resonance of the BA2PbI4 film.

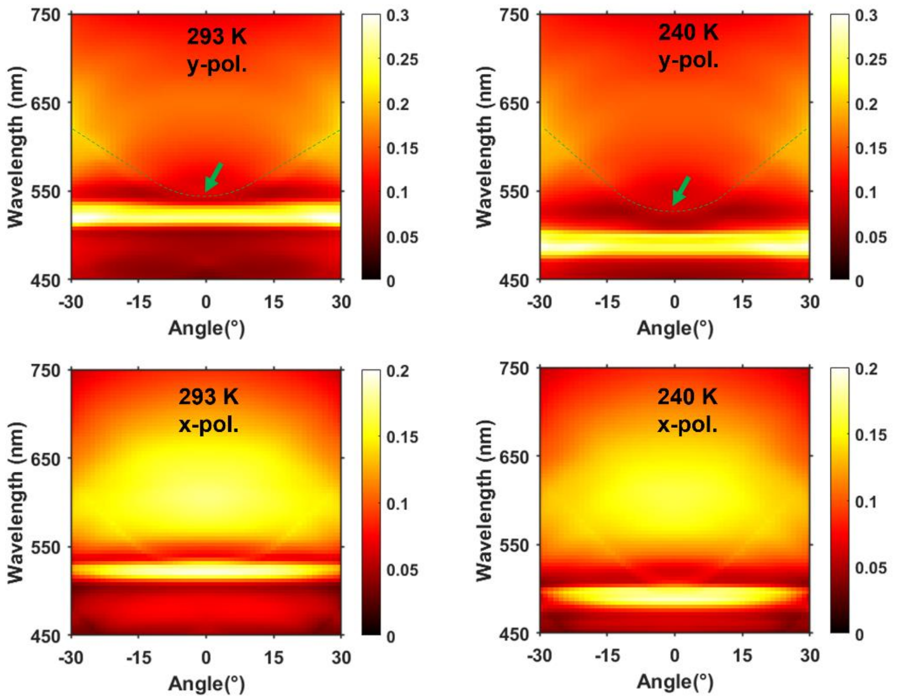

Fig. S5 The calculated angle-resolved reflection map at room temperature and 240 K for both x and y-polarized illuminations.
